# A Novel Method (CiMON) for Continuous Intra-Abdominal Pressure Monitoring: Pilot Test in a Pig Model

**DOI:** 10.1155/2012/181563

**Published:** 2012-02-20

**Authors:** Joost Wauters, Liesbeth Spincemaille, Anne-Sophie Dieudonne, Kenny Van Zwam, Alexander Wilmer, Manu L. N. G. Malbrain

**Affiliations:** ^1^Medical Intensive Care Unit, University Hospital Gasthuisberg, Herestraat 49, 3000 Leuven, Belgium; ^2^Intensive Care Unit, Ziekenhuisnetwerk Antwerpen, Campus Stuivenberg, 2060 Antwerpen, Belgium

## Abstract

*Background*. Intravesical pressure (IAP_ivp_) measurement is considered to be the gold standard for assessment of intra-abdominal pressure (IAP). This study evaluated a new minimally invasive IAP monitoring device (CiMON) against three other devices in a wide range of clinically relevant IAP and in different body positions in healthy pigs. *Methods*. The CiMON catheter (IAP_CiM_) and another balloon-tipped catheter (IAP_spie_) were positioned into the stomach. Fluid-filled catheters were used for direct intraperitoneal (IAP_dir_) and IAP_ivp_ measurement. Both in supine and 25° head-of-bed positions, IAP was increased from baseline to 30 mmHg. At every IAP level, 4 IAP measurements were recorded simultaneously. Mean differences and the limits of agreement were calculated. *Results*. Bias between IAP_CiM_ and IAP_spie_ was nearly zero with very good agreement, both in supine and 25° position. In supine position, IAP_CiM_ slightly overestimated IAP_ivp_ and IAP_dir_ by 1.5 and 2.1 mmHg with reasonable agreement. In 25° position, IAP_CiM_ underestimated IAP_ivp_ and IAP_dir_ by 1.0 and 0.5 mmHg, again with reasonable agreement. *Conclusions*. Agreement between IAP_CiM_ and IAP_spie_ was very good, while good-to-moderate agreement exists between IAP_CiM_ and IAP_dir_ or IAP_ivp_. Simplicity, continuous monitoring, and the combination with a feeding tube should lead to further clinical studies, evaluating this new CiMON device.

## 1. Introduction

Intra-abdominal hypertension (IAH) and abdominal compartment syndrome (ACS) have significant morbidity and mortality [1–3]. IAH is defined as a sustained elevated intra-abdominal pressure (IAP) ≥ 12 mmHg without organ dysfunction, and ACS is defined as a sustained IAP > 20 mmHg associated with new organ dysfunction. The definitions of IAH and ACS are thus based on the determination of intra-abdominal pressure (IAP), measured at end-expiration in the complete supine position after ensuring that abdominal muscle contractions are absent [4]. Although IAP measured intermittently via the bladder (with an instillation volume of maximal 25 mL sterile saline) is the current standard of care to screen patients for IAH/ACS, this technique has important limitations. Several contraindications for bladder catheterization exist (e.g., pelvic trauma and urinary tract infection). Moreover, bladder measurements are still mostly intermittent in nature, being labour intensive and possibly preventing timely recognition of significant changes in IAP [5]. Some fully automated continuous monitoring techniques have been described and validated in vitro and in animal laboratories [6–8]. Several of these more invasive techniques perform very well in laboratory settings but are less feasible in clinical scenarios. Transgastric devices might be a reasonable minimally invasive alternative for continuous IAP monitoring [9–13]. Recently, a new transgastric device became available that uses an air-filled balloon positioned on a nasogastric feeding tube [14]. The aim of the present study was to evaluate this new device against three other IAP measurement devices (direct intraperitoneal, bladder, and gastric) in a wide range of clinically relevant IAP and in different body positions with regard to precision and applicability in a future human investigation.

## 2. Materials and Methods

### 2.1. Animal Instrumentation

**Figure 1:**
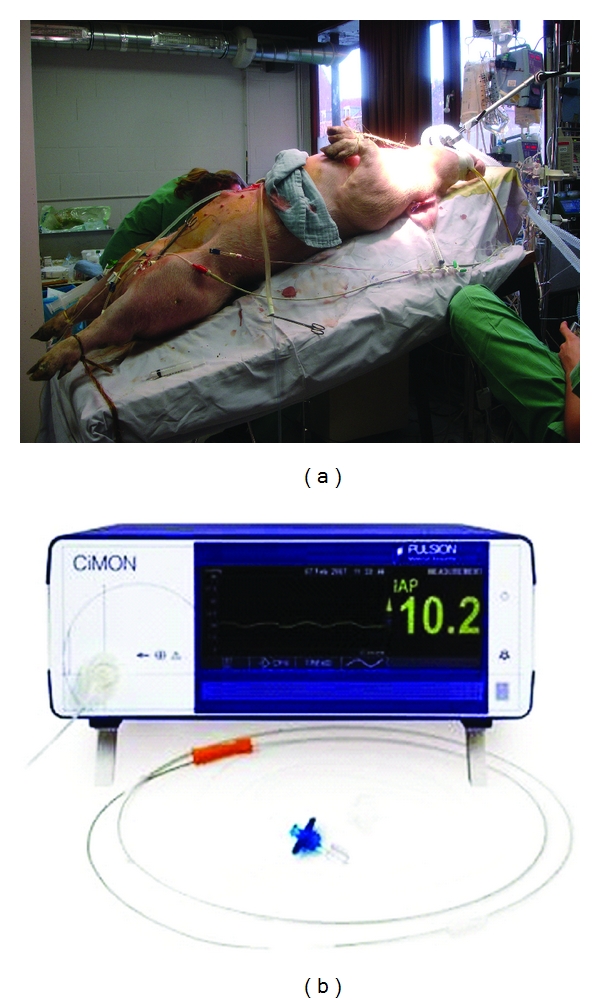
(a) Pig setup in head of bed (HOB) 25° in animal lab at Leuven University. (b) CiMON monitor and probe with feeding lumen.

This study was performed in accordance with the national guidelines for ethical animal research and was approved by the local Institutional Ethics Committee on Animal Care and Use. After overnight fasting, 6 anaesthetized and paralyzed pigs (mean body weight of 39 ± 1 kg) were mechanically ventilated (Evita XL, Drager, Lubeck, Germany) using an oxygen concentration (FiO2) of 35%, a tidal volume (TV) of 9 mL/kg with an inspiration/expiration ratio of 1/2 and a PEEP of 7 cmH_2_0. Respiratory rate was adjusted to maintain arterial paCO_2_ between 35–45 mmHg. These settings were kept constant throughout the experiment. A femoral artery catheter was inserted for blood pressure monitoring and blood gas sampling. Ringer lactate at 4 mL·kg^−1^·h^−1^ (Viaflex, Baxter, Lessines, Belgium) and HAES-steril at 5 mL·kg^−1^·h^−1^ (FreeFlex, Fresenius, Friedberg, Germany) were administered via a femoral vein catheter. The pigs were instrumented with 4 different IAP measurement catheters. Two balloon-tipped catheters (IAP_spie_ and IAP_CiM_) were positioned transesophageally into the stomach. Their position was checked afterwards by radioscopy. A small midline laparatomy was performed and a fluid-filled catheter (IAP_dir_) was placed intra-peritoneally, caudally to the stomach. A standard Foley catheter (IAP_ivp_) was inserted into the bladder. All catheters were exteriorised, and the laparotomy was carefully closed water-sealed in two layers.  Figure 1(a) shows the pig setup.

### 2.2. Measurements of IAP

Measurements of IAP were based on 4 different measurement principles.

**Spiegelberg Balloon-Tipped Catheter IAP Measurement (IAP_spie_)**

This IAP measurement device (Spiegelberg, Hamburg, Germany) has been described previously and consists of a nasogastric tube-like catheter (outer diameter 3 mm) equipped with an air-filled balloon (total filling volume 1 mL) connected to a device for automatic zeroing, control, and pressure measurement [7, 15].

**CiMON Balloon-Tipped Catheter IAP Measurement (IAP_CiM_)**

The CiMON system (Pulsion Medical Systems, Munich, Germany) consists of a nasogastric probe (outer diameter 5.3 mm) with a small air-inflatable balloon (total filling volume 1.1 mL) located at the distal tip of the probe (Figure 1(b)). The probe has one lumen that connects the air-filled balloon with the IAP monitor and one feeding lumen that can also be used for guide wire introduction. The balloon is connected to a device for automatic zeroing, control, and pressure measurement.

**Intravesical Pressure IAP Measurement (IAP_ivp_)**

Before each measurement, the bladder was emptied. An instillation volume of 20 mL saline was injected to measure intravesical pressure [4]. Twenty milliliter is in accordance with the research recommendations of the World Society on Abdominal Compartment Syndrome (WSACS, http://www.wsacs.org/), and it was well below the average volume of fluid needed to increase the intravesical pressure by 2 mmHg in pigs with an IAP of 20 mmHg [16, 17]. The 10 Ch multiple-hole fluid-filled catheter (Cystofix, BBraun, Melsungen, Germany) was connected to a pressure transducer. The level of the symphysis pubis was taken as zero reference, both with the pig supine and in 25° head elevation.

**Direct Intra-Peritoneal IAP Measurement (IAP_dir_)**

The multiple-hole fluid-filled catheter was connected to a two-way stopcock. One side was continuously flushed (5 mL/h) to prevent obstructions in the catheter lumen. The other side was connected to a pressure transducer and the symphysis pubis was taken as zero reference, both with the pig supine and in 25° head elevation.

### 2.3. Experimental Protocol

The pig being in supine position, IAP was increased from baseline up to 20 and 30 mmHg (as measured by the intravesical IAP) by infusing warmed saline intraperitoneally. At each IAP-level, 2 times 4 continuous IAP-traces (2 quadruplets) were recorded simultaneously for 5 minutes (after a stabilization period of 5 minutes). Then, IAP was decreased again to 20 mmHg and baseline, and measurements were repeated. Then, the pig was placed in 25° head of bed position. Similarly, IAP was increased from baseline up to 30 mmHg and back to baseline and IAP-traces were recorded. In each position, a horizontally calibrated radioscopy was performed to measure vertical height differences between the different catheter tips. At the end of the experiment, animals were sacrificed by hypertonic potassium chloride injection under deep anaesthesia.

### 2.4. Data Acquisition and Data Analysis

IAP_dir_ and IAP_ivp_ were captured by a multimodal monitor (Philips IntelliVue, Best, The Netherlands) connected to a computer for real-time data saving via a LAN. The Spiegelberg device (IAP_spie_) was connected via a serial port (50 Hz) to another computer for real-time data saving. The CiMON device (IAP_CiM_) was connected to a third computer via a serial port (50 Hz). The internal clocks of the 3 different computers were synchronized before starting data acquisition. Afterwards, time-synchronized IAP data from different devices were analyzed off line with dedicated software (Matlab 6.5.1, MathWorks, USA, and Trendface Solo 1.1.5, Ixellence, Hamburg, Germany). Since IAP_dir_ was always zeroed at the level of the symphysis pubis, IAP_dir_ was corrected for the height of the fluid column between the intraperitoneal measurement point and the pressure transducer. From every IAP quadruplet, we calculated 3 different IAP data pairs (IAP_CiM_−IAP_spie_, and IAP_CiM_−IAP_ivp_, IAP_CiM_−IAP_dir_) and corrected them for the vertical height difference between the catheter tips, as measured with horizontally calibrated radioscopy. All IAP values were measured end-expiratory and are expressed in mmHg.

### 2.5. Statistical Analysis

Results are expressed as mean ± standard deviation (SD = precision). According to the method of Bland Altman, bias was calculated as the mean difference between values obtained with different measurement techniques, and limits of agreement were calculated as mean ± 2SD [17]. Percentage error was calculated as the limits of agreement divided by the mean IAP. Based on the recent WSACS recommendations, good agreement between 2 techniques was defined as follows: bias ≤ 1 mmHg, limits of agreement between −4 to 4 mmHg, and a maximal percentage error of 25% [18]. 

## 3. Results

**Figure 2:**
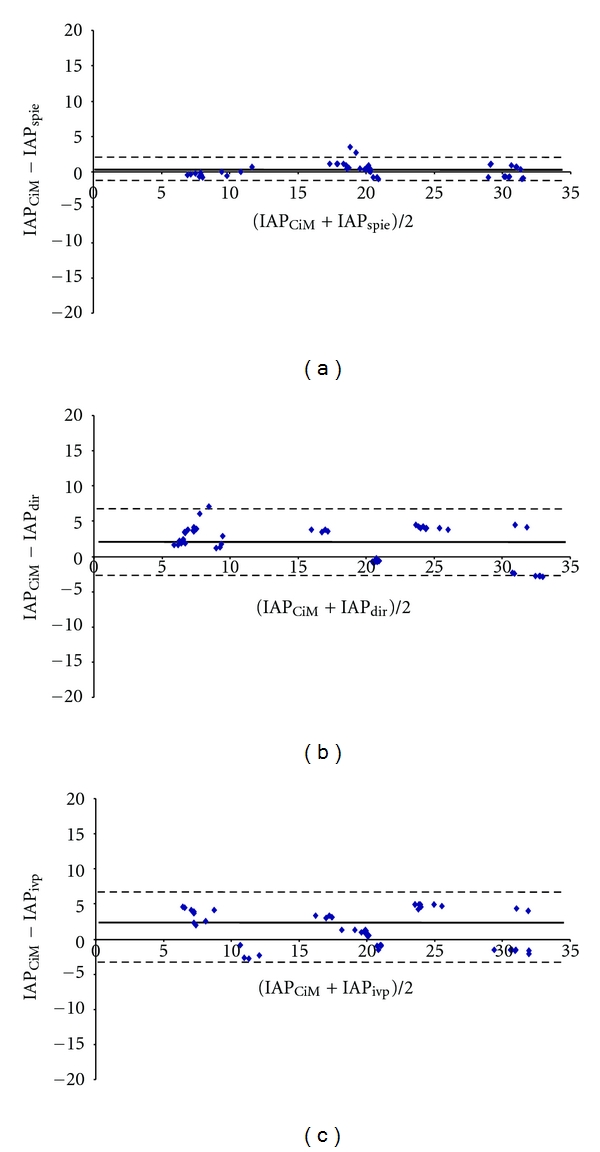
Bland Altman plots for comparing different methods measuring intra-abdominal pressure (IAP) in supine position. *Horizontal full line:* bias; *horizontal dashed line:* upper and lower limits of agreement. (a) CiMON balloon-tipped catheter IAP measurement (IAP_CiM_) versus Spiegelberg balloon-tipped catheter IAP measurement (IAP_spie_). (b) IAP_CiM_ versus direct intraperitoneal IAP measurement (IAP_dir_). (c) IAP_CiM_ versus intravesical pressure IAP measurement (IAP_ivp_).

**Figure 3:**
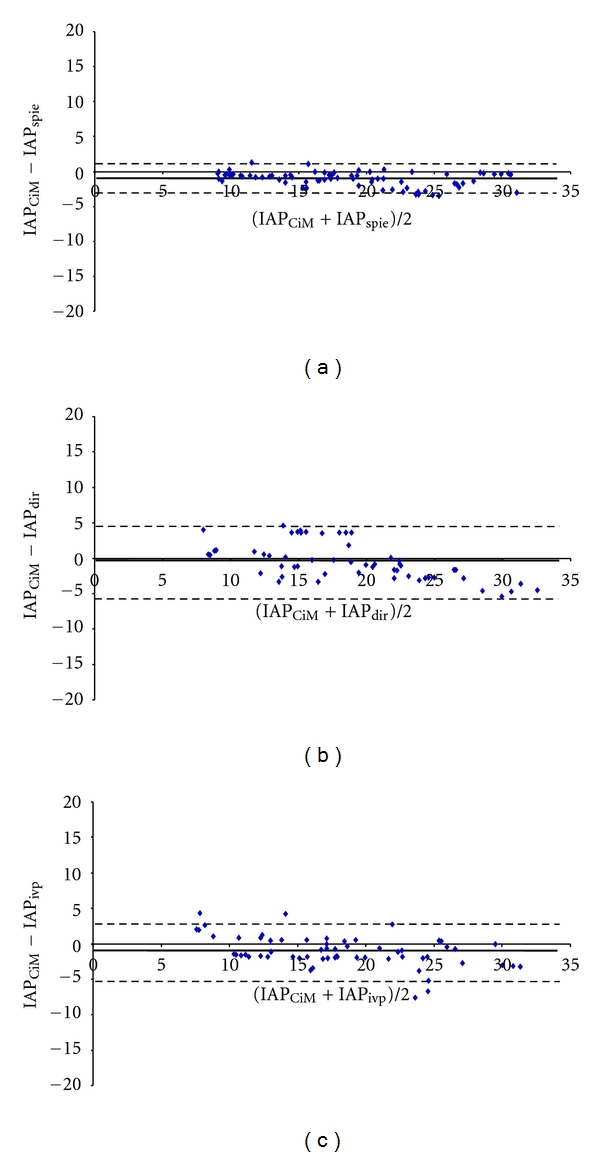
Bland Altman plots for comparing different methods measuring intra-abdominal pressure (IAP) in 25° head-of-bed position. *Horizontal full line:* bias; *horizontal dashed line:* upper and lower limits of agreement. (a) CiMON balloon-tipped catheter IAP measurement (IAP_CiM_) versus Spiegelberg balloon-tipped catheter IAP measurement (IAP_spie_). (b) IAP_CiM_ versus direct intraperitoneal IAP measurement (IAP_dir_). (c) IAP_CiM_ versus intravesical pressure IAP measurement (IAP_ivp_).

The CiMON probe was successfully inserted transesophageally in all pigs. A total of 528 IAP measurements (248 in supine and 280 in 25° head-up elevation position) were used to calculate agreement between the different devices, resulting in 62 IAP quadruplets in supine and 70 IAP quadruplets in 25° head-up elevation position. In supine position, 10 quadruplets (40 IAP measurements), and in 25° position, 2 quadruplets (8 IAP measurements) dropped out due to technical reasons. Baseline IAP, measured intraperitoneally, was 3.9 ± 1.9 mmHg. IAP ranged from 2.6 to 31.2 mmHg (average 15.2 mmHg) in supine position and from 7.0 to 40.4 mmHg (average 22.1 mmHg) in 25° position. In the supine position, the CiMON and the bladder catheter were almost on the same height (difference 0.7 cm), while at 25° head elevation, the CiMON probe was 7.5 cm higher than the bladder catheter. Bias between IAP_CiM_ and IAP_spie_ was nearly zero with very good agreement. IAP_CiM_ overestimated IAP_ivp_ and IAP_dir_ by 1.5 and 2.1 mmHg, respectively with reasonable agreement. Data on precision, percentage error and limits of agreement between different techniques in supine position are presented in Table 1 and Figure 2. In 25° head-up elevation, there was also very good agreement between the intragastric IAP measurements (CiMON and Spiegelberg). IAP_CiM_ underestimated IAP_ivp_ and IAP_dir_ by 1.0 and 0.5 mmHg, respectively, again with reasonable agreement. Mean differences between different device readings, precision, percentage error, and limits of agreement in the 25° head-up elevation position are presented in Table 2 and Figure 3.

## 4. Discussion 

This study evaluated a new intragastric IAP measurement device (CiMON) against three other IAP measurement devices (gastric, direct, and bladder) in a wide range of clinically relevant IAP and in different body positions. 

First of all, we validated the new CiMON device against another balloon-tipped intragastric IAP measurement technique (Spiegelberg). This device has already been evaluated as a valid tool for IAP measurement [9, 10]. As expected, we found very good agreement between these two intragastric techniques, both in supine and in 25° head-up elevation positions. Although calibration-triggered volume expansion of one balloon was captured by the other balloon, significant interference between both IAP measurements was negligible. Second, reasonable agreement was found between CiMON and intravesical IAP measurements. Bias was not more than 1.5 mmHg with a percentage error less than 25% in both supine and 25° anti-Trendelenburg position, and limits of agreement were not above 6 mmHg. Similar agreement was obtained when CiMON data was evaluated against directly measured IAP. Our results are comparable with data from others, evaluating several intragastric devices for IAP measurement [11–13]. Comparing gastric with intravesical IAP in patients undergoing laparoscopic cholecystectomy, Sugrue et al. found limits of agreement of −4 to +3 mmHg with a bias of 0.35 mmHg (IAP range from 8 to 20 mmHg) [11]. Turnbull et al. compared gastric with directly measured IAP in 29 patients undergoing elective laparoscopy, both in supine an 15° head-down position [13]. Bias was nearly zero, and limits of agreement were −2.5 to 4.5 mmHg in supine and −4.6 to 2.4 mmHg in Trendelenburg position. De Waele et al. found that IAP measured using an intragastric compliance catheter reliably reflects the reference IAP in seven patients undergoing laparoscopic cholecystectomy [19]. In these studies, the air-filled experimental setup allowed the authors to neglect height differences. In our study, we used intra-abdominal fluid to generate IAP, better mimicking the clinical situation. Indeed, in critically ill patients, IAH and ACS are often induced by fluid accumulation such as ascites, generalized edema, paralytic ileus, or hemoperitoneum. Therefore, we measured hydrostatic height differences and corrected for them. However, measuring hydrostatic height differences in clinical practice is time consuming and not straightforward. This again stresses the need for standardisation of IAP measurements in the supine position, as stated by the consensus definitions of the WSACS, making the comparison in the supine position the most important in this study [4, 17]. 

This new CiMON device has several advantages but some questions remained unanswered. The risk for infection is negligible, nearly all ICU patients need a nasogastric feeding tube in place, and probe position is often checked by radioscopy. CiMON measurement and zeroing is fully automated, saving time and labour intensive actions. The CiMON device monitors continuous IAP, allowing IAP and IAP-derived data trends such as abdominal perfusion pressure (= mean arterial pressure − IAP) to be used to treat critically ill patients [20–23]. However, the effects of gastric migratory motor complexes, enteral feeding, or administration of prokinetics on IAP measurements are not known [24]. Piessevaux et al. found erythromycine to result in fundic pressure wave changes of 20 mmHg in conscious healthy volunteers [25]. Yet, these temporary perturbations might become irrelevant when continuous IAP monitoring is done. Further studies are necessary to answer these questions. 

In conclusion, this study evaluated a new transgastric IAP measurement device (CiMON) against three other IAP measurement devices (direct, bladder, and gastric) in a wide range of clinically relevant IAP and in different body positions. Agreement between both intragastric balloon-tipped devices was very good, while agreement between CiMON and direct or intravesical IAP measurement was reasonable. The advantages of simplicity, continuous monitoring possibilities and the combination with a feeding tube should lead to further clinical studies, evaluating this new device.

## Figures and Tables

**Table 1:** Statistics of comparison between different device readings in the supine position.

Device	Bias (mmHg)	Precision (mmHg)	Limits of agreement (mmHg)	Percentage error
IAP_CiM_−IAP_spie_	0.2	0.9	−1.6 to 2.0	9.6
IAP_CiM_−IAP_dir_	2.1	2.5	−2.9 to 7.1	31.6
IAP_CiM_−IAP_ivp_	1.5	2.5	−3.5 to 6.6	24.9

Mean difference between different device readings (bias), precision (standard deviation), percentage error (%), and limits of agreement in the supine position. CiMON balloon-tipped catheter pressure (IAP_CiM_), Spiegelberg balloon-tipped catheter pressure (IAP_spie_), direct intraperitoneal pressure (IAP_dir_), and intravesical pressure (IAP_ivp_) measurement.

**Table 2:** Statistics of comparison between different device readings in the 25° head-up elevation position.

Device	Bias (mmHg)	Precision (mmHg)	Limits of agreement (mmHg)	Percentage error
IAP_CiM_−IAP_spie_	−1.1	1.1	−3.2 to 1.0	11.0
IAP_CiM_−IAP_dir_	−0.5	2.7	−5.8 to 4.8	26.3
IAP_CiM_−IAP_ivp_	−1.0	2.2	−5.4 to 3.3	19.9

Mean difference between different device readings (bias), precision (standard deviation), percentage error (%), and limits of agreement in the 25° head-up elevation position. CiMON balloon-tipped catheter pressure (IAP_CiM_), Spiegelberg balloon-tipped catheter pressure (IAP_spie_), direct intraperitoneal pressure (IAP_dir_), and intravesical pressure (IAP_ivp_) measurement.

## Abbreviations

IAP: Intra-abdominal pressure
IAP_ivp_: Intravesical pressure
IAP_CiM_: CiMON balloon-tipped catheter intra-abdominal pressure
IAP_spie:_: Spiegelberg balloon-tipped catheter intra-abdominal pressure
IAP_dir_: Direct intraperitoneal pressure
IAH: Intra-abdominal hypertension
ACS: Abdominal compartment syndrome
FiO2: Inspired oxygen fraction
TV: Tidal volume
PEEP: Positive end-expiratory pressure
paCO_2_: Arterial carbon dioxide partial pressure
WSACS: World Society of Abdominal Compartment Syndrome
SD: Standard deviation
ICU: Intensive care unit.
